# Encapsulation of Hydrophobic Apigenin into Small Unilamellar Liposomes Coated with Chitosan Through Ethanol Injection and Spray Drying

**DOI:** 10.1007/s11947-023-03140-y

**Published:** 2023-06-13

**Authors:** San-San Ang, Yin Yin Thoo, Lee Fong Siow

**Affiliations:** School of Science, Monash University Malaysia, 47500 Subang Jaya, Selangor Malaysia

**Keywords:** Apigenin, Liposomes, Chitosan, Spray drying, Encapsulation

## Abstract

Despite the multiple health benefits, natural flavonoid apigenin has poor aqueous solubility that restricts its delivery in foods. This study investigated the potential of spray-dried chitosan-coated liposomes prepared from scalable methods for the food industry as the delivery carriers for apigenin. Apigenin-loaded small unilamellar liposomes produced from ethanol injection had an encapsulation efficiency of 74.88 ± 5.31%. They were electrostatically stabilised via chitosan coating (0.25% w/v) and spray-dried. Spray-dried chitosan-coated apigenin liposomes (SCAL) exhibited the following powder characteristics: yield 66.62 ± 3.08%, moisture content 4.33 ± 0.56%, water activity 0.2242 ± 0.0548, particle size 10.97 ± 1.55 μm, nearly spherical morphology with wrinkles and dents under microscopic observation. Compared with the unencapsulated apigenin, SCAL demonstrated improved aqueous solubility (10.22 ± 0.18 mg/L), higher antioxidant capacity, and stability against simulated gastrointestinal digestion. The chitosan coating gave a slower in-vitro release of apigenin in SCAL (77.0 ± 6.2%) than that of uncoated apigenin liposomes (94.0 ± 5.3%) at 12 h. The apigenin release kinetics from SCAL could be represented by the Korsmeyer-Peppas model (R^2^ = 0.971). These findings suggest that SCAL could be a promising delivery system of apigenin for functional food applications.

## Introduction

Nowadays, consumers actively seek functional foods that could provide health and wellness benefits beyond essential nutrition. This trend has been accelerated due to the global Covid-19 pandemic, where consumers are taking more proactive measures than ever to improve their health and prevent diseases (Askew, 2021). Plant flavonoids have long been regarded as valuable antioxidants that can be used as therapeutic agents, preventatives, and for maintaining human health. Apigenin (4’, 5, 7-trihydroxyflavone) is a flavonoid present in parsley, chamomile flower, and celery. It has been reported with a broad spectrum of pharmacological properties, which include antioxidant, anti-inflammatory, anti-cancer, anti-diabetic, and anti-depression effects (Liang et al., 1999; Nakazawa et al., 2003; Nielsen et al., 1999; Piantelli et al., 2006; Ren et al., 2016). Given the numerous health benefits of apigenin, the dietary intake of the compound through foods may greatly interest the health-conscious market. However, the poor aqueous solubility of apigenin could probably lead to its low bioaccessibility and bioavailability when delivered through food matrices which are mostly hydrophilic (Zhang et al., 2012).

In recent years, there has been a growing interest in exploring the potential of various encapsulation methods for hydrophobic flavonoids. Many studies have been published in this area, highlighting the effectiveness of encapsulation in preserving and delivering these compounds in food systems. For instance, researchers have successfully encapsulated luteolin in nanostructured lipid carriers (Liu et al., 2014), hesperidin in polysaccharide hydrogel particles (Tsirigotis-Maniecka et al., 2017), rutin in oil-in-water emulsion (Dammak & do Amaral Sobral, 2017), naringin in zein/caseinate biopolymers (Nallamuthu et al., 2020), and quercetin in inorganic Ca_3_(PO_4_)_2_ nanofiber (Mojtabavi et al., 2023). Notably, liposomal encapsulation has emerged as a promising method for encapsulating hydrophobic flavonoids owing to the amphiphilic nature of liposomes that facilitates the incorporation of poorly aqueous soluble compounds into water-based systems. This encapsulation strategy has been found effective for flavonoids including quercetin (Hao et al., 2017), naringenin (Wang et al., 2017), and rutin (Lopez-Polo et al., 2020). Additionally, liposomes offer a safe alternative as they possess a bilayer structure of phospholipids that closely resemble biological cell membranes, making them biocompatible and thereby reducing the risk of possible toxic effects (Premathilaka et al., 2022; Shah et al., 2020).

Previous studies have demonstrated the potential of using liposomes to encapsulate substances for food applications. For example, thyme extract liposomes were incorporated into whey protein isolate films as an antimicrobial agent in food packaging (Aziz & Almasi, 2018); vitamin D liposomes were used to fortify yoghurt (Jafari et al., 2019); brown algae extract liposomes had been explored as natural antioxidant and antimicrobial agents in mayonnaise (Savaghebi et al., 2021); myrtle extract loaded in liposomes had demonstrated improved antioxidant and antimicrobial properties as a promising food preservative (Gorjian et al., 2022); nisin liposomes were incorporated into active composite packaging to extend the shelf life of chicken breast filets and cheese slices (Niaz et al., 2022). While liposomal encapsulation for hydrophobic flavonoids like quercetin (Hao et al., 2017; Román-Aguirre et al., 2020; Souza et al., 2014; Toniazzo et al., 2017) and rutin (Lopez-Polo et al., 2020; Sengupta et al., 2023; Silva-Weiss et al., 2018) has been well studied for food purposes, there is currently limited reports on apigenin liposomes in this context. Previous studies on apigenin liposomes have primarily focused on their therapeutic potential (Banerjee et al., 2017; Jin et al., 2017; Sen et al., 2019; Shen et al., 2014). Given that apigenin is a dietary flavonoid with the potential to be used as a functional food ingredient, further research is required to determine the feasibility of using apigenin liposomes for food applications, which could pave the way for the development of functional foods.

On the other hand, ethanol injection is deemed a highly efficient and straightforward approach for producing a homogenous population of small unilamellar liposomes without needing high-energy steps such as sonication and homogenisation (Gouda et al., 2021; Liu et al., 2020a). This method is desirable for scaling up liposomes compared to the thin film hydration method used in previous liposomal studies (Azarashkan et al., 2022; Jafari et al., 2019; Pinilla et al., 2020; Souri et al., 2023). In light of the current emphasis on industrial sustainability and energy efficiency, it is worth exploring the feasibility of using ethanol injection for producing apigenin liposomes, especially since there is a lack of information on this particular topic within the existing literature. Moreover, the thin film hydration method often uses hazardous solvents such as chloroform, methanol, and acetone, which can raise concerns about food safety. On the contrary, ethanol is generally recognised as safe (GRAS), making ethanol injection a safer and more suitable option for producing liposomes for food applications. To date, the research on food-related apigenin liposomes (Paini et al., 2015; Zhang et al., 2020) has been quite limited, and the important aspects for food applications such as the aqueous solubility, antioxidant capacity, and in-vitro activities of apigenin encapsulated in liposomes, have not been adequately addressed.

Furthermore, liposomes are thermodynamically unstable and prone to aggregation during storage; therefore biopolymers such as alginate (Liu et al., 2016), chitosan (Zhou et al., 2018), whey protein isolate (Frenzel et al., 2015), inulin (Román-Aguirre et al., 2020), and basil seed gum (Azarashkan et al., 2022) had been explored as liposomal coatings to improve their stability. Among these, chitosan, a natural polysaccharide derived from the chitin of crustacean shells, is the most extensively used due to its biocompatibility, biodegradability, and low toxicity. Through electrostatic interaction, the coating of positively-charged chitosan provides a steric stabilisation effect on the liposomes (Esposto et al., 2021). In addition, chitosan coating also prolongs the in-vitro release of encapsulated compounds from liposomes and provides their stability against gastrointestinal digestion (Tai et al., 2020; Zhou et al., 2021). Although a previous similar study had demonstrated the stability of encapsulated quercetin in chitosan-coated nano-liposomes (Souza et al., 2014), there is currently a lack of information on the in-vitro activities and gastrointestinal stability of hydrophobic flavonoids encapsulated in small unilamellar liposomes.

Meanwhile, recent efforts have been made to remove the water content from liposomal suspension by means of spray drying, freeze drying or lyophilisation, thereby allowing for long-term storage and increased versatility. For instance, spray-dried yoghurt powder fortified with vitamin D liposomes (Jafari et al., 2019) and freeze-dried liposomes loaded with garlic extract and nisin (Pinilla et al., 2020) were successfully produced and showed potential for food use. While freeze drying requires longer processing time, higher capital, and operating costs, spray drying is preferred in the food industry due to its low cost, simplicity, and high efficiency (Buljeta et al., 2022; Karthik & Anandharamakrishnan, 2013). Recent studies have also explored the spray drying of chitosan-coated liposomes encapsulating hydrophilic phenolic-rich extracts and protein hydrolysates (Altin et al., 2018; Guldiken et al., 2019; Ma et al., 2022; Sarabandi & Jafari, 2020). However, there is currently limited research on the potential of spray-dried chitosan-coated liposomes in encapsulating hydrophobic flavonoids. Additionally, the study about spray-dried chitosan-coated liposomes for apigenin has not been reported before.

Considering the aforementioned knowledge gaps, this study aims to evaluate the potential of spray-dried chitosan-coated liposomes as delivery carriers of apigenin for food applications. Using ethanol injection, small unilamellar liposomes encapsulating apigenin were prepared, coated with chitosan, and characterised (particle size distribution, zeta potential, encapsulation efficiency, morphology). The chitosan-coated apigenin liposomes were then spray-dried, followed by the determination of their physicochemical characteristics and powder morphology. The aqueous solubility, antioxidant capacity, in-vitro release profile, and stability against gastrointestinal digestion of encapsulated apigenin were also evaluated.

## Materials and Method

### Materials

Apigenin (AP) with 97.95% purity was purchased from TargetMol Chemicals Inc. (MA, USA). Soy lecithin (> 94% phosphatidylcholine), cholesterol (≥ 99%), chitosan (low molecular weight, ≥ 75% degree of deacetylation), Sephadex^®^ G-50-Fine, 2,2′-azobis (2-methylpropionamidine) dihydrochloride (AAPH), 2,2′-Azino-bis(3-ethylbenzothiazoline-6-sulfonic acid) diammonium salt (ABTS), Folin-Ciocalteu’s phenol reagent, Trolox, gallic acid, fluorescein sodium salt, pepsin (porcine, ≥ 500 U/mg), pancreatin (porcine, 4 X USP), and bile extract porcine were supplied by Sigma-Aldrich (Missouri, USA). Tween 80, maltodextrin (10–12 DE), iron (III) chloride, acetonitrile (HPLC grade), hydrochloric acid, and sodium chloride were purchased from Chemiz (M) Sdn. Bhd. (Malaysia). Methanol, sodium carbonate, trichloroacetic acid, and potassium persulfate were obtained from Fisher Chemicals (MA, USA). Other chemicals used include ethanol absolute (VWR International Ltd, UK), isopropanol (Systerm Chemicals, Malaysia), iron (II) sulfate, potassium chloride, and dipotassium hydrogen phosphate (R&M Chemicals, Malaysia). All chemicals used were of analytical grade unless specified. Water purified from Milli-Q system (Millipore, USA) was used in all analyses.

### Preparation of Uncoated Liposomes

Apigenin liposomes (AL) were prepared by ethanol injection method as described by Sebaaly et al. (2015) with modification. The weight ratio of the liposomal ingredients was determined according to the previous suggestion to give small-sized liposomes (Schubert & Müller-Goymann, 2003; Shaker et al., 2017; Zou et al., 2014), whereas the apigenin concentration (2% of soy lecithin weight) was proposed by Zhang et al. (2020) for high encapsulation efficiency. Briefly, soy lecithin (0.40 g), Tween 80 (0.08 g), cholesterol (0.04 g), and AP (0.008 g) were dissolved in 20 mL of ethanol absolute. Using a syringe, the ethanolic mixture was injected into 60 mL of acetate buffer (pH 3.6) under magnetic stirring (500 rpm). The mixture was continuously stirred for 15 min at room temperature for liposome formation. After that, the ethanol in the suspension was removed by rotary evaporation (Eyela N-1100 VW, Tokyo Rikakikai Co.Ltd, Japan) at 50 °C for 10 min. Lastly, the volume was adjusted to 80 mL using acetate buffer before filtration through a 0.22 μm nylon membrane. Control liposomes (CL) without AP were prepared using a similar method. AL and CL, each in triplicates, were stored at 4 °C in the dark.

### Preparation of Chitosan-coated Liposomes

A preliminary study revealed that 0.25% w/v chitosan in the suspension stabilised liposomes after 3 h of coating process. First, 0.50% w/v chitosan solution was prepared in acetate buffer pH 3.6. Then, AL was added dropwise to the chitosan solution in a 1:1 volume ratio to achieve 0.25% w/v chitosan in the suspension. The mixture was gently stirred (200 rpm) at room temperature for 3 h. The chitosan-coated apigenin liposomes (CAL) and chitosan-coated control liposomes (CCL) were stored at 4 °C in the dark for further analyses (Hao et al., 2017).

### Characterisation of Uncoated and Chitosan-coated Liposomes

#### Particle Size Distribution, Polydispersity Index, and Zeta Potential

The particle size distribution, polydispersity index (PDI), and zeta potential of liposomes were measured by dynamic light scattering on a Zetasizer (Nano-ZS, Malvern Instruments, UK) at 25 °C. Samples were diluted with water at a volume ratio of 1:9 to eliminate multiple scattering phenomena. The refractive index was set at 1.33. The zeta potential measurement was performed using Smoluchowski’s mathematical model with a clear disposable zeta cell on the same instrument (Zhang et al., 2020).

#### Transmission Electron Microscopy (TEM)

The appearance of AL and CAL was examined by TEM (FEI Tecnai G2 Transmission Electron Microscope, Thermo Scientific, USA) with sodium tungstate (1% w/v, pH 6.6) as the negative staining (Melchior et al., 1980). AL and CAL were diluted 1:10 with acetate buffer before placing them on a carbon-coated copper grid. After drying at room temperature, the grid was stained and dried before TEM observation at an accelerating voltage of 200 kV.

#### Determination of Encapsulation Efficiency (EE) by HPLC

A Sephadex gel mini-column was used to remove the unencapsulated AP from the AL suspension. Hydrated Sephadex (0.40 g dry Sephadex in 10 mL water) was transferred into a 5 mL syringe to form a gel column of approximately 3 cm height. Acetate buffer (1.5 mL) was added to the gel, and the column was centrifuged at 1000 rpm for 3 min. Next, 1.5 mL of sample was added, and the centrifugation was repeated (Gültekin-Özgüven et al., 2016). The gel-filtered sample collected was treated with ethanol absolute to quantify AP on HPLC. The chromatography was performed on a C18 reverse-phase column (Zorbax Eclipse Plus C18, 4.6 × 250 mm, 5 μm) with isocratic elution of water and acetonitrile in the ratio 40:60 (v/v). The setting was as follows: flow rate 1 ml/min, column temperature 30 °C, injection volume 10 μL, UV detection wavelength 337 nm (Li et al., 1997). The retention time of standard AP was observed at 2.90 min. The EE of AP was calculated using Eq. (1) (Zhang et al., 2020).

1 $$EE\left(\%\right)= \frac{{C}_{2}}{{C}_{1} }\times 100$$

where *C*_*1*_ is the concentration of AP added in the liposomal formulation, and *C*_*2*_ is the concentration of encapsulated AP recovered from gel filtration.

### Spray Drying

Chitosan-coated liposomes containing 10% w/v maltodextrin and 0.25% w/v chitosan were prepared in acetate buffer as the spray-dry feed. After stirring for 30 min, the feed was spray dried on a Büchi B-290 Mini Spray Dryer (Büchi Labortechnik, Flawil, Switzerland) equipped with a 0.7 mm diameter nozzle tip. The spray drying parameters were as follows: inlet temperature 120 °C, feed rate 2.5 mL/min, gas flow 667 L/h, aspiration rate 35 m^3^/h. The spray-dried chitosan-coated apigenin liposomes (SCAL) and spray-dried chitosan-coated control liposomes (SCCL) were stored in airtight containers and kept at 4 °C for further analyses (Guldiken et al., 2019).

### Characterisation of Spray-dried Chitosan-coated Liposomes

#### Powder Yield

The powder yield of spray drying was calculated from the total weight of dry powder collected and the raw materials added to the feed solution (Goëlo et al., 2020).

2 $$Powder\ yield \left(\%\right)= \frac{Total\ weight\ of\ dry\ powder\ collected}{Total\ weight\ of\ raw\ materials\ in\ feed\ solution}\times 100$$

#### Moisture Content and Water Activity

The moisture content of spray-dried liposomes was examined on a halogen moisture analyser (HR-83 Halogen, Mettler Toledo, Switzerland). At the same time, their water activity was measured using a water activity meter (AquaLab 4TE, Decagon Devices, USA).

#### Particle Size Distribution

The particle size distribution of spray-dried liposomes was analysed by a laser light diffraction analyser (Malvern Mastersizer 3000, Malvern Panalytical Ltd., UK) equipped with a Hydro EV wet disperser. The powder was dispersed in isopropanol until an obscuration level of 5-10%. The size measurement was carried out on Mie diffraction theory with refractive indexes of 1.56 (particle) and 1.39 (dispersant), rotating speed of 1000 rpm, and 60% ultrasound level (Xu et al., 2019).

#### Powder Morphology

The morphology of spray-dried liposomes was examined using a field emission scanning electron microscope (FE-SEM, Hitachi SU 8010, Tokyo, Japan). The powders were mounted on an aluminum stub using double-sided carbon adhesive tape. The stub was then sputtered with a thin layer of gold at 30 mA for 35 s under vacuum (Quorum, Q150R ES, UK). The SEM analysis was performed with an accelerating voltage of 5 kV at high vacuum.

### Aqueous Solubility Analysis

Referring to the method described by Telange et al. (2017) with minor modification, SCAL (0.50 g) and unencapsulated AP (0.27 mg) were added to 30 mL of water and shaken overnight (100 rpm) at room temperature. The aqueous phase at the top was mixed with methanol to solubilise AP before centrifugation at 5000 rpm for 15 min. The supernatant was subjected to HPLC analysis described previously in the section of “Determination of Encapsulation Efficiency (EE) by HPLC” to examine the concentration of AP in the aqueous phase.

### Antioxidant Capacity

To examine the antioxidant capacity, SCAL was reconstituted by dissolving 0.5 g powder in 4.5 ml of acetate buffer. The reconstituted SCAL was then treated with methanol to dissolve the chitosan layer and vesicles, followed by centrifugation at 12000 rpm for 30 min. At an equivalent concentration of AP (~27 mg/L), SCAL was compared to unencapsulated AP and AL, as well as with SCCL.

#### Folin-Ciocalteu Assay

The total phenolic content (TPC) was examined using the Folin-Ciocalteu method described previously with slight modifications (Dag & Oztop, 2017). Samples (0.5 mL) were added to 2.5 mL of diluted Folin-Ciocalteu reagent (1:10 v/v in water) and mixed well using a vortex. After a 3-min incubation in the dark, 2 mL of sodium carbonate (7.5% w/v) was added. The sample was left to stand in the dark for 1 h, followed by centrifugation (1000 rpm, 3 min) before measuring its absorbance at 760 nm on a UV–vis spectrophotometer (Lambda 365 UV–vis spectrophotometer, PerkinElmer Instruments Inc., USA). The TPC of the sample was expressed as milligram gallic acid equivalent per L sample (mg GAE/L).

#### ABTS Radical Scavenging Assay

The ABTS radical scavenging assay was performed according to the method of Xiao et al. (2020) with minor modifications. Acetate buffer (pH 4.5) was used to prepare the ABTS radical reaction solution, which contained ABTS (7 mM) and potassium persulfate (2.45 mM) in an equal volume ratio. After overnight incubation at room temperature in the dark, the solution was diluted into ABTS radical working solution with spectrophotometric absorbance of ~0.700 at 734 nm. Sample (0.5 mL) was added to 3.5 mL of ABTS working solution, and the absorbance at 734 nm was recorded after 1 h of incubation at 37 ºC. The ABTS radical scavenging activity was expressed as mg Trolox equivalent per L sample (mg TE / L).

#### Oxygen Radical Absorbance Capacity (ORAC) Assay

The ORAC assay was carried out in black opaque 96-well microplates at 37 °C as described by Ho et al. (2017) with minor modifications. Phosphate buffer (75 mM, pH 7.4) was used as the blank, while Trolox (10–100 μM) was used as the standard. In each well, 150 μL of sodium fluorescein (81.6 nM in phosphate buffer) was mixed with 25 μL of sample or Trolox solution. After incubation for 10 min at 37 °C in a microplate reader (Tecan, 200 Pro, USA), 25 μL of AAPH (153 mM in phosphate buffer) was added, followed by plate shaking for 10 s. The fluorescence was recorded every 60 s for 90 cycles at excitation and emission wavelength of 485 nm and 528 nm. The area under the fluorescence decay curve (AUC) and net AUC for samples were calculated using Eqs. (3) and (4) (Wannenmacher et al., 2019). The ORAC value was expressed as uM Trolox equivalent (μM TE).

3 $$AUC=1+\frac{{f}_{2}}{{f}_{1}}+ \frac{{f}_{3}}{{f}_{1}}+\dots +\frac{{f}_{90} }{{f}_{1}}$$

4 $$Net\ AUC=AU{C}_{sample }-AU{C}_{blank}$$

where *f*_*1*_ is the fluorescence value at cycle 1, *f*_*90*_ is the fluorescence value at cycle 90.

#### Ferric Reducing Antioxidant Power (FRAP) Assay

The ferric reducing power was evaluated according to the method of Tan et al. (2014) with modifications. Briefly, the mixture of 0.5 mL of sample and 0.5 mL of potassium ferricyanide (2.5% w/v) was incubated at 50 °C for 20 min. Then, 2.5 mL of trichloroacetic acid (10% w/v) was added, followed by centrifugation at 5000 rpm for 2 min. The supernatant (1 mL) was mixed with 2 mL of water and 0.5 mL of ferric chloride (0.1% w/v). After incubation at 50 °C for 10 min, the absorbance of the mixture at 700 nm was measured. The FRAP value of the sample was expressed as mM Fe^2+^.

### In-vitro Analysis

#### In-vitro Release Profile

The in-vitro release study was conducted in pH 7 to simulate intestinal physiological condition (Haznar-Garbacz et al., 2022). Firstly, SCAL, AL, and unencapsulated AP with similar AP concentrations (~27 mg/L) were prepared in 10 mL phosphate buffer pH 7 and placed in dialysis tubing (MCWO 12–14 kDa). The dialysis tubing was then immersed into 30 mL phosphate buffer added with 0.50%w/v Tween 80 and 10% v/v ethanol as dissolution medium. The samples were incubated at 37 °C and 100 rpm. At predetermined intervals, 1 mL of aliquot was withdrawn from the dissolution medium and subsequently replaced with 1 mL of dissolution medium. The AP concentration was measured on HPLC, and the cumulative release of AP was calculated using Eq. (5) (Zhou et al., 2021).

5 $$Cumulative\ release\ \left(\%\right)=\frac{Cumulative\ released\ AP\ in\ dissolution\ medium}{Initial\ amount\ of\ AP}\times 100$$

#### Release Kinetics

To understand the release mechanism of AP from SCAL, the cumulative release data collected were fitted to the following models. The best-fitted model was selected based on the highest correlation coefficient, R^2^ (Liu et al., 2020b).

6 $$\begin{array}{cc}\mathrm{First\ order}& C=kt\end{array}$$

7 $$\begin{array}{cc}\mathrm{Second\ order}& \mathrm{ln}\left(1-C\right)=-kt\end{array}$$

8 $$\begin{array}{cc}\mathrm{Higuchi}& C=k{t}^{1/2}\end{array}$$

9 $$\begin{array}{cc}\text{Korsemeyer-Peppas}& C=k{t}^{n}\end{array}$$

where *C* is the cumulative released AP at time *t*, *k* is the release rate constant, and *n* is the diffusion coefficient representing the drug release mechanism.

#### Stability Against Simulated Gastrointestinal Digestion

The simulated gastrointestinal digestion was carried out as described previously (Chi et al., 2019; Tai et al., 2020). Saline solution (1.6 g/L NaCl, 0.20 g/L KCl), simulated gastric fluid/SGF (3.2 g/L pepsin, 2 g/L NaCl, 7 mL/L concentrated HCl), and simulated intestinal fluid/SIF (3.2 g/L pancreatin, 5 g/L bile extract, 8.8 g/L NaCl, 6.8 g/L K_2_HPO_4_) were prepared. SCAL, AL, and unencapsulated AP (with AP concentration of ~27 mg/L) were added to 5 mL of saline solution. For gastric digestion, 5 mL of SGF was added and the pH was adjusted to 2 before 2 h of incubation at 37 °C and 100 rpm. After that, the pH was adjusted to 6.5–7.0 using 1 M NaOH before adding a similar volume of SIF for intestinal digestion. After 2 h of incubation, the mixture was immediately placed in ice bath to stop the enzymatic reaction. The concentration of AP in samples at before and after digestion was measured by HPLC, while the antioxidant activity was analysed by the ABTS assay described earlier.

## Results and Discussion

### Characterisation of Uncoated and Chitosan-coated Liposomes

Nanoparticles, according to the European Food Safety Authority (EFSA), are defined as engineered nanomaterials that are less than 100 nm in size. For drug delivery, nanoparticles or nanoscale carriers are desirable due to their larger surface area that could lead to higher permeability and reactivity, ultimately enhances drug delivery efficacy. Liposomes with a size range of 100–150 nm are considered ideal for achieving prolonged circulation time in the bloodstream, as they are less susceptible to rapid renal filtration (which affects 10–15 nm particles) or clearance by the mononuclear phagocyte system (which affects > 150 nm particles) (Premathilaka et al., 2022).

Table 1 summarises the average size, PDI, and zeta potential of uncoated (CL, AL) and coated liposomes (CCL, CAL). CL and AL showed the characteristics of small unilamellar liposomes (≤ 100 nm), with an average size of 92.46–95.21 nm (Liu et al., 2020a). PDI is an important parameter for measuring size distribution of nanoparticles, with PDI value of 0.0 indicates a sample with perfectly uniform particles, while a PDI value of 1.0 indicates a sample with polydisperse particles. A PDI values of 0.3 and less represents a relatively homogenous sample (Danaei et al., 2018). The PDI values of 0.141–0.144 obtained for the liposomes suggests that they are highly homogenous with less risk of aggregation. These results show that it is possible to achieve a highly monodisperse distribution (PDI < 0.3) of nano-sized liposomes using ethanol injection method without using sonication or homogenisation step. The literature has reported similar findings for liposomes prepared from ethanol injection, such as α-tocopherol liposomes (118 ± 13 nm, PDI = 0.155 ± 0.019) and caffeine liposomes (80–95 nm, PDI = 0.12 – 0.17) (Charcosset et al., 2015; Pham et al., 2012). However, chitosan coating at 0.25% w/v led to an increase in the average size and PDI values of CCL and CAL (p < 0.05). Nonetheless, the chitosan-coated liposomes remained within the desirable size of 100–150 nm for drug delivery and maintained a homogenous distribution (PDI < 0.3).

**Table 1:** Average size, PDI, and zeta potential of uncoated and coated liposomes

**Sample**	**Average size (nm)**	**PDI**	**Zeta potential (mV)**
CL	92.46 ± 3.09^a^	0.141 ± 0.011^a^	3.61 ± 0.13^a^
AL	95.21 ± 0.25^a^	0.144 ± 0.006^a^	3.83 ± 1.06^a^
CCL	128.78 ± 9.67^b^	0.289 ± 0.009^b^	34.97 ± 3.88^b^
CAL	138.92 ± 10.01^b^	0.290 ± 0.019^b^	34.20 ± 1.87^b^

Data represent mean ± standard deviation (n = 3). Values with different superscript letters within the columns are significantly different (p < 0.05)

Zeta potential, which measures the electric potential at the interface between charged particles and dispersants, is an indicator of colloidal stability: highly unstable (±0–10 mV), relatively stable (±10–20 mV), moderately stable (±20–30 mV), and highly stable (> ± 30 mV) (Bhattacharjee, 2016). CL and AL were considered highly unstable (< 10 mV), which may lead to particle aggregation over time. With the deposition of chitosan with positively-charged amino groups on the liposomal surface, CCL and CAL had higher positive zeta potential over 30 mV (p < 0.05), indicating that the liposomes could be stabilised by the adequate electrostatic repulsion effect (Mertins & Dimova, 2011). On the other hand, previous studies observed negative zeta potential in their uncoated liposomes (Sebaaly et al., 2015; Zou et al., 2014), in contrast to the positive zeta potential observed in this study. This is possibly due to the difference in the phosphatidylcholine (PC) content in the lecithin and the buffer used compared to previous studies. As PC has 0 mV at pH 4, lecithin with higher PC content tends to give a positively-charged liposomal surface when dispersing in an acidic buffer with a high concentration of H^+^ ions (Naumowicz et al., 2013).

**Fig. 1:**
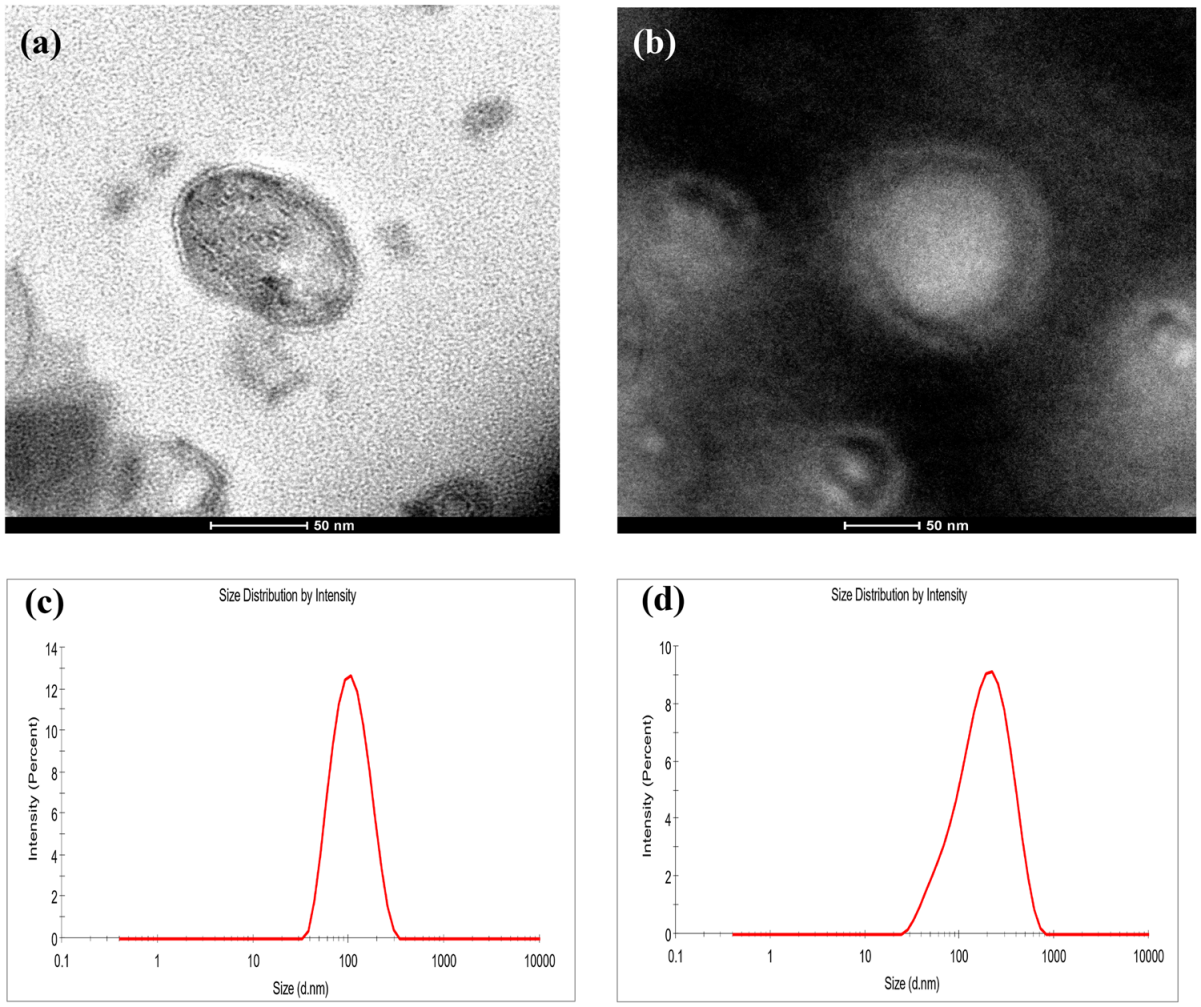
TEM images of AL (**a**), CAL (**b**) and particle size of AL (**c**) and CAL (**d**) measured by dynamic light scattering. AL: apigenin liposomes; CAL: chitosan-coated apigenin liposomes

From the TEM images (Fig. 1a, b), AL and CAL were nearly spherical and unilamellar. The liposomes had a diameter in the nanometer range under microscopic observation, which confirms the particle size results obtained from dynamic light scattering (Fig. 1c, d). With chitosan coating, CAL appeared larger with a visible chitosan layer surrounding the liposomes. A similar TEM morphology was noted for uncoated and gum arabic-coated green tea extract liposomes prepared from ethanol injection by Dag et al. (2019).

As small-sized unilamellar liposomes do not tend to settle at the bottom to form a pellet, Sephadex gel filtration is recommended to remove the unencapsulated compounds rather than centrifugation for the EE analysis (Betageri, 1996). Unencapsulated compounds with low molecular weight are retained in the Sephadex porous beads made of cross-linked dextran, while liposomes are eluted from the gel column. The EE of AP into small unilamellar liposomes was found to be 74.88 ± 5.31%, which is similar to those reported for apigenin liposomes prepared from the thin-film method (~75%) (Zhang et al., 2020) and high-pressure homogenisation (80.18%) (Shetti & Jalalpure, 2021). This suggests that the ethanol injection method is an effective way to encapsulate AP into liposomes without compromising the EE. Moreover, the EE value obtained for our liposomes is also comparable to that reported for garlic extract liposomes (82.1 – 85.2%), and higher than the EE recorded for brown algae extract liposomes (45.5 ± 1.2%), which were prepared using the thin film method and Mozafari method, respectively (Pinilla et al., 2020; Savaghebi et al., 2021).

### Characterisation of Spray-dried Chitosan-coated Liposomes

Table 2 shows the powder yield and physicochemical properties of spray-dried chitosan-coated liposomes. The powder yield from the spray drying of chitosan-coated liposomes was 66.62 – 71.03%. At the same feed rate (2.5 mL/min), chitosan-coated liposomes encapsulating black carrot extract, back mulberry extract, and ghrelin hormone reported spray dry yield of 45.9 ± 4.26%, 66.0 – 75.1% and 87.4%, respectively (Guldiken et al., 2019; Gültekin-Özgüven et al., 2016; Salade et al., 2018). The powder yield obtained for chitosan-coated liposomes with apigenin liposomes (66.62 ± 3.08%) in this study falls within the range of the mentioned studies. Additionally, yoghurt fortified with vitamin D liposomes had a similar spray dry yield of 73.88% at the same maltodextrin concentration (Jafari et al., 2019). However, it is worth noting that the powder yield obtained in this study is higher than other encapsulation systems reported. For example, the powder yield of curcumin encapsulated in gum arabic and propolis loaded in protein matrices ranged from 29–42% and 20.09–52.67%, respectively (Bucurescu et al., 2018; Jansen-Alves et al., 2018). The variation in the spray dry yield may be due to differences in the encapsulation system, spray-drying conditions, or the properties of the encapsulating agents used (Shamaei et al., 2017).

**Table 2:** Powder yield and physicochemical properties of spray-dried chitosan-coated liposomes

**Sample**	**Powder yield** **(%)**	**Moisture content** **(%)**	**Water activity** **(A**_**w**_**)**	**Particle size, D**_**50**_ **(µm)**
SCCL	71.03 ± 1.74^a^	4.13 ± 0.36^a^	0.2098 ± 0.0019^a^	11.50 ± 0.46^a^
SCAL	66.62 ± 3.08^a^	4.33 ± 0.56^a^	0.2242 ± 0.0548^a^	10.97 ± 1.55^a^

Data represent mean value ± standard deviation (n = 3). Values with different superscript letters within the columns are significantly different (p < 0.05). The powder yield and physicochemical properties of SCCL and SCAL were not significantly different

**Fig. 2:**
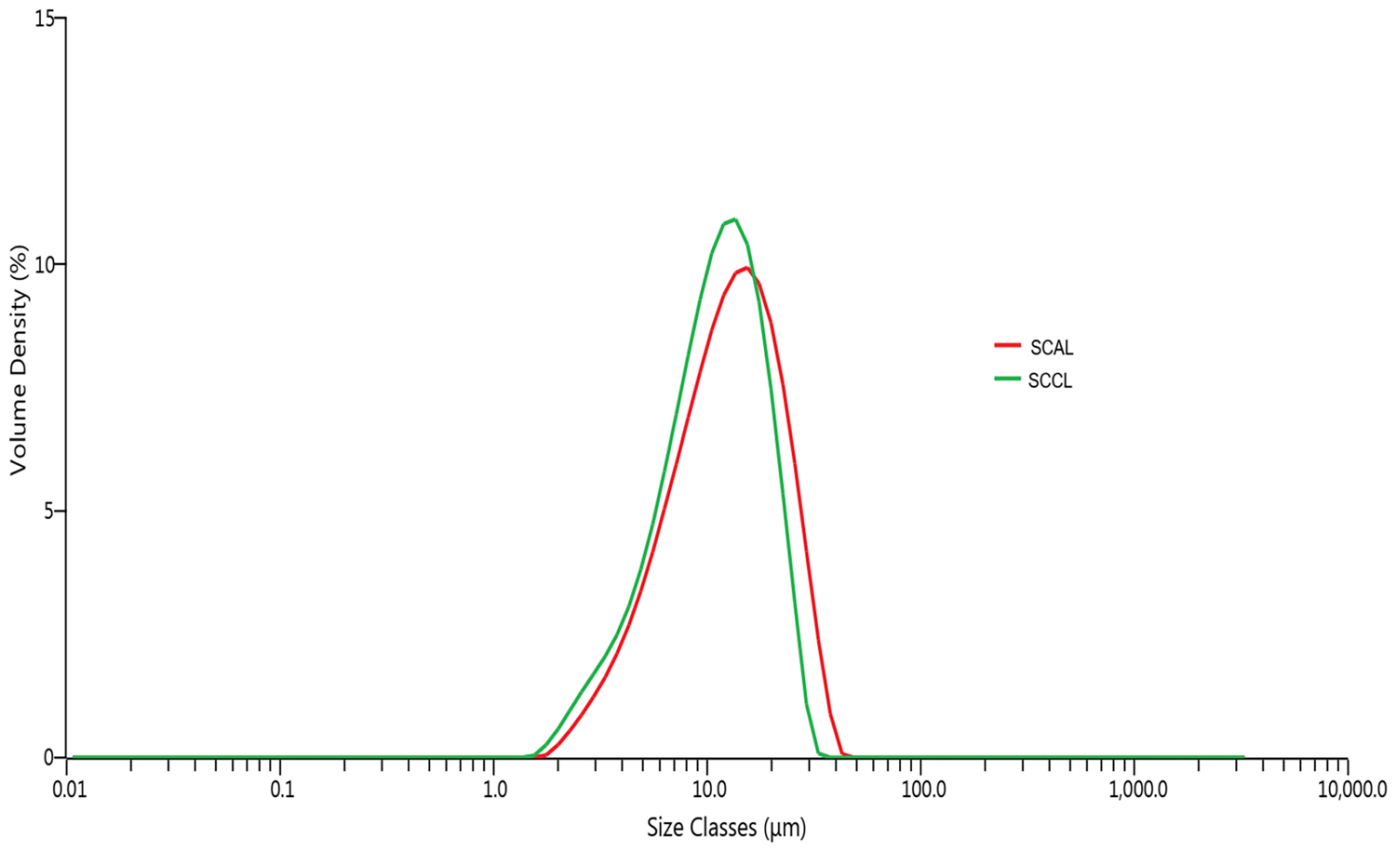
Particle size distribution of SCAL and SCCL. SCAL: spray-dried chitosan-coated apigenin liposomes; SCCL: spray-dried chitosan-coated control liposomes

The moisture content (< 10%) and water activity (< 0.60) of SCAL and SCCL indicate that the liposomes had been adequately dried at the selected inlet temperature and feed rate for microbial safety and storage stability (Vera Zambrano et al., 2019). On the contrary, previous studies have shown that freeze-dried liposomes can have higher residual moisture levels exceeding 10%, which may affect the powder characteristics and quality during storage (Marín et al., 2018; Pinilla et al., 2020). As shown in Fig. 2, both SCCL and SCAL had a narrow particle size distribution, with their D_50_ around 11 μm. Different particle size was reported previously for black carrot extract (16.9 ± 0.3 μm; 20% maltodextrin) and flaxseed-peptide fractions (8.4 ± 0.6 μm; 5% maltodextrin), possibly due to the different concentration of maltodextrin in the feed (Guldiken et al., 2019; Sarabandi & Jafari, 2020).

**Fig. 3:**
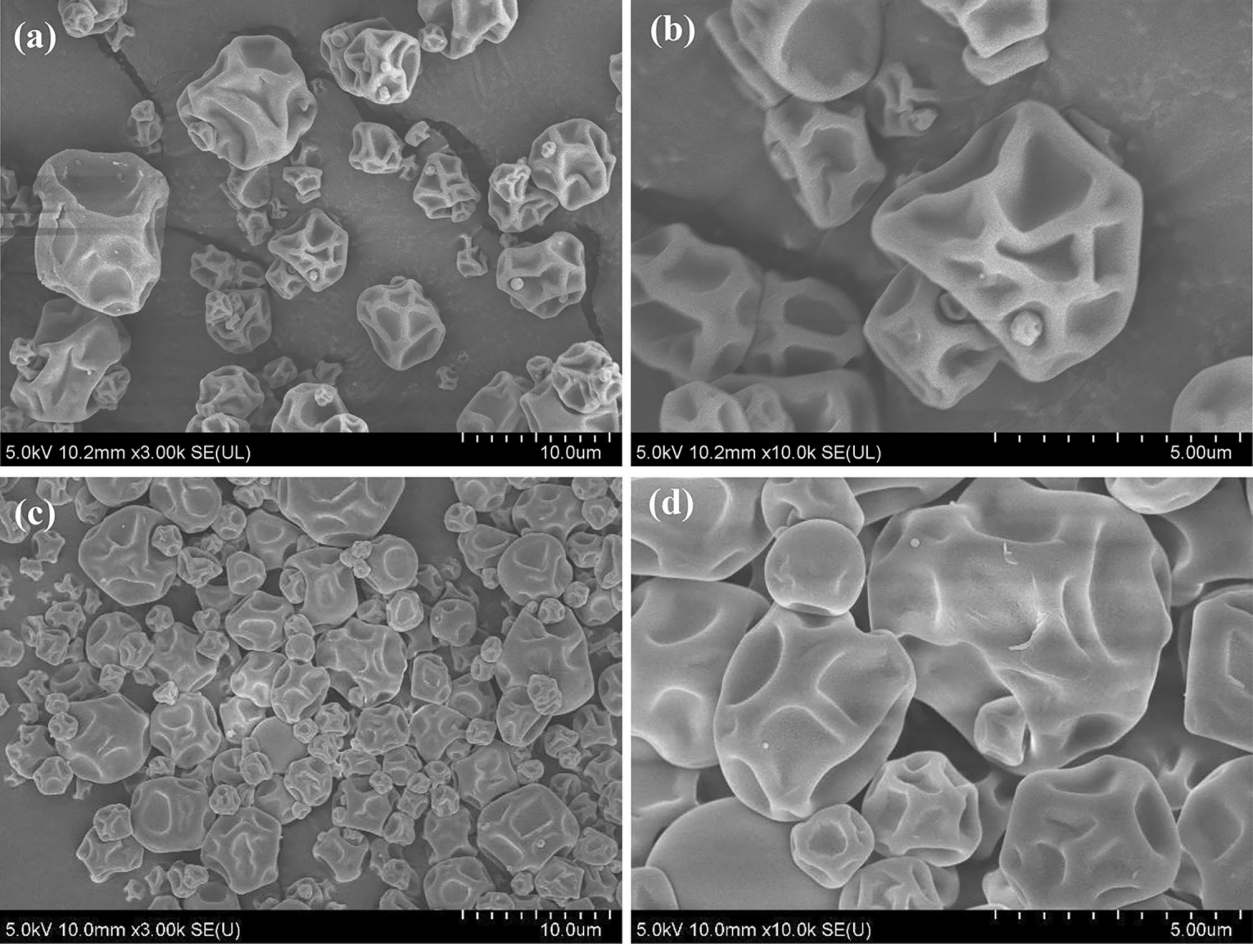
SEM images of SCAL (**a**, **b**) and SCCL (**c**, **d**) at 3000× and 10000× magnification. SCAL: spray-dried chitosan-coated apigenin liposomes; SCCL: spray-dried chitosan-coated control liposomes

As shown in the SEM images (Fig. 3), SCCL and SCAL are nearly spherical, with diameters ranging from 5 μm to 10 μm, which agrees with the result of the aforementioned particle size. Since spherical particles have reduced surface contact with the surrounding environment, they provide a higher degree of controlled release and better protection of encapsulated compounds (Sarabandi & Jafari, 2020). Wrinkled and dents were observed on the powder surface, similar to the previous studies (Akgün et al., 2020; Altin et al., 2018; Guldiken et al., 2019; Gültekin-Özgüven et al., 2016). A high evaporation rate of spray drying leads to an earlier crust formation on the droplet surface. Water vapour inside the crust may undergo thermal expansion, rupturing it and causing the particle to deflate and shrivel, making wrinkles and dents appear (Karadag et al., 2013).

### Aqueous Solubility

AP in its pure form has very poor aqueous solubility: 1.35 µg/mL in water (Li et al., 1997) and 1.63 μg/mL in phosphate buffer pH 7 (Zhang et al., 2012). It has been suggested that the double bonds of flavones (position 2 and 3) can form a planar structure, creating a tight molecular arrangement that impedes the penetration of solvent molecules and result in their poor aqueous solubility (Zhao et al., 2019). Owing to the amphiphilicity of liposomes, AP encapsulated in SCAL exhibited a significant increase in aqueous solubility compared to the unencapsulated AP (Table 3). The improved AP aqueous solubility had also been reported when the compound was encapsulated in phytosomes (37-fold higher) and 2-hydroxypropyl-β-cyclodextrin (> 40-fold higher) in previous studies (Telange et al., 2017; Wu et al., 2017).

**Table 3:** Aqueous solubility of unencapsulated AP and SCAL

**Sample**	**Solubility (mg/L)**
Unencapsulated AP	n.d.
SCAL	10.22 ± 0.18

Data represent mean value ± standard deviation (n = 3)

*n.d.* not detected

### Retention of AP and its Antioxidant Capacity during Spray Drying

It is crucial to retain the encapsulated compounds during spray drying to ensure their biological and antioxidant activity in powder. As shown in Table 4, there was no significant difference between the concentration and antioxidant activity (in terms of TPC) of AP in SCAL before and after spray drying (p > 0.05). The good retention of AP against the spray dry temperature of 120 °C could be supported by a previous finding on the thermal stability of AP at 100 °C water bath for 5 h (Hostetler et al., 2013). Conversely, at higher inlet temperatures of 140–150 °C, previous studies found that the antioxidant activity of encapsulated compounds was less retained. Spray drying of chitosan-coated liposomes with cacao hull waste extract showed 39% retention of TPC (Altin et al., 2018), while 69.23 ± 5.1% retention of TPC was reported for black mulberry waste extract (Gültekin-Özgüven et al., 2016).

**Table 4:** Concentration of AP and total phenolic content (TPC) in SCAL

**Sample**	**AP concentration (mg/L)**	**TPC (mg GAE/L)**
Before spray drying	28.82 ± 1.00^a^	163.05 ± 9.96^a^
After spray drying	27.07 ± 3.82^a^	174.16 ± 10.58^a^

Data represent mean value ± standard deviation (n = 3). Values with different superscript letters within the columns are significantly different (p < 0.05). No significant difference was observed in the AP concentration and TPC between both samples

### Antioxidant Capacity

**Fig. 4:**
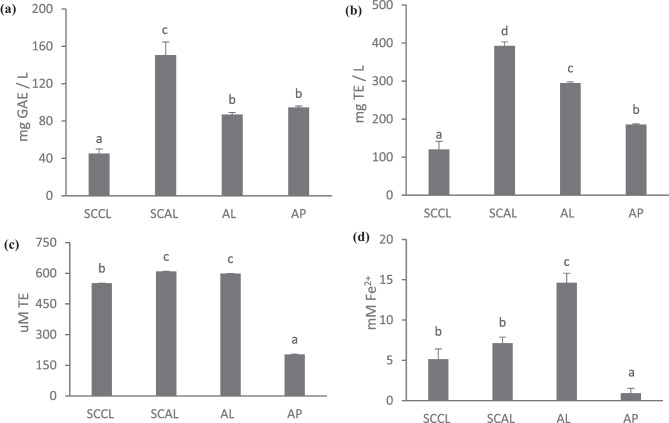
Antioxidant capacity of SCCL, SCAL, AL, and AP measured by Folin Ciocalteau (**a**), ABTS (**b**), ORAC (**c**), and FRAP (**d**) assays. Different lowercase letters indicate significant differences at p < 0.05 (n = 3). SCCL: spray-dried chitosan-coated control liposomes; SCAL: spray-dried chitosan-coated apigenin liposomes; AL: apigenin liposomes; AP: unencapsulated apigenin

The antioxidant capacity of SCCL, SCAL, AL, and unencapsulated AP was examined using the suggested in-vitro assays in the literature (Kashyap et al., 2022). ABTS and ORAC assays measure radical scavenging activity according to the electron transfer (ET) and hydrogen transfer (HT) reactions, respectively. Folin-Ciocalteau assay determines the total antioxidant capacity from total phenolic content (TPC), whereas FRAP assay measures reducing power. As presented in Fig. 4, SCAL had the highest TPC, ABTS and ORAC values, at 150.71 ± 13.87 mg GAE/L, 392.51 ± 10.53 mg TE/L, and 608.74 ± 11.11 μM TE, respectively. Interestingly, despite the absence of AP, SCCL exhibited antioxidant capacity in all the four assays. Phosphatidylcholine and chitosan have been reported with antioxidant activity (Liu et al., 2020b; Pinilla et al., 2020; Wan et al., 2013). As a result of the synergistic antioxidant capacity, SCAL showed remarkably higher antioxidant capacity than the unencapsulated AP across all the assays and higher TPC and ABTS values than AL (p < 0.05). Increased antioxidant activity had also been reported earlier for spirulina hydrolysate encapsulated in chitosan-coated liposomes compared to its unencapsulated counterpart (Mohammadi et al., 2023).

### In-vitro Release and Release Kinetics

**Fig. 5:**
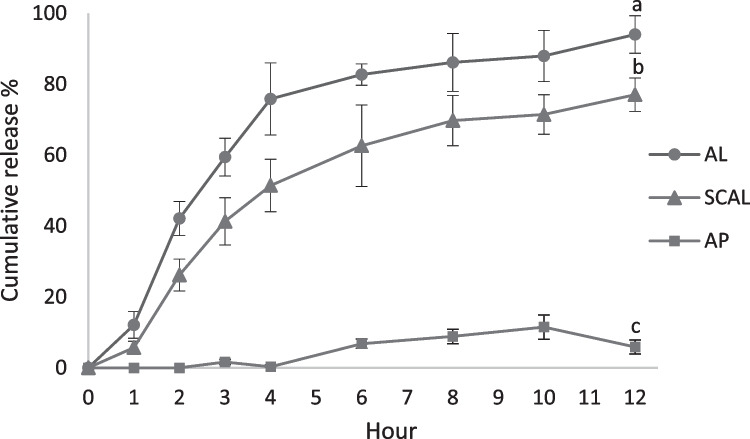
Cumulative release profile of AL, SCAL, and AP over 12 h of analysis. Different lowercase letters indicate significant differences at p < 0.05 (n = 3). AL: apigenin liposomes; SCAL: spray-dried chitosan-coated apigenin liposomes; AP: unencapsulated apigenin

Figure 5 shows the in-vitro release profiles of SCAL, AL, and unencapsulated AP at 37 °C in phosphate buffer pH 7. Overall, AL demonstrated a more rapid release rate than SCAL over the test period. At 12 h, higher AP release was observed from uncoated AL (94.0 ± 5.3%) than SCAL (77.0 ± 6.2%) (p < 0.05). In SCAL, the external chitosan layer could have played a role in prolonging the release of AP from liposomes. The sustained release effect of chitosan coating has been demonstrated in curcumin and acteoside liposomes (Liu et al., 2015; Zhou et al., 2018). Meanwhile, unencapsulated AP in crystal form showed poor release from dialysis bags, possibly because of its poor wettability and solubility. Pure AP was found with a similar release pattern in previous studies (Sarim et al., 2022; Telange et al., 2017).

The kinetic equations of cumulative AP release from SCAL are presented in Table 5, along with their respective correlation coefficients (R^2^) when fitted to the models. Korsmeyer-Peppas had the highest R^2^, suggesting that SCAL’s release data fit the model best. A similar release model was also reported for quercetin and itraconazole encapsulated in chitosan-coated liposomes previously (Colino et al., 2022; Rizal et al., 2021). Furthermore, the diffusion coefficient (*n*) of this model can be used to describe the primary mechanism of release: Fickian diffusion (*n* ≤ 0.45), non-Fickian anomalous transport (0.45 < *n* < 0.89), and super Case II transport (*n* > 0.89) (Zhao & Wang, 2019). As indicated by the value of *n* obtained (0.574), the release of AP from SCAL is a non-Fickian anomalous transport process, which involves the synergistic effect of diffusion and dissolution of chitosan and liposomal phospholipid layer (Liu et al., 2020b).

**Table 5:** Model fitness of the AP release profile from SCAL

**Model**	**Equation**	***K***	***R***^***2***^	***n***
Zero order	C = 0.064t + 0.1237	0.064	0.857	-
First order	ln (1-C) = -0.126t - 0.081	-0.126	0.953	-
Higuchi	C = 0.253t^−0.056^	0.253	0.942	-
Korsmeyer-Peppas	C = 0.199t^0.574^	0.199	0.971	0.574

*K* release rate constant, *R*^*2*^ correlation coefficient, *n* diffusion coefficient

### Stability Against Gastrointestinal Digestion

**Fig. 6:**
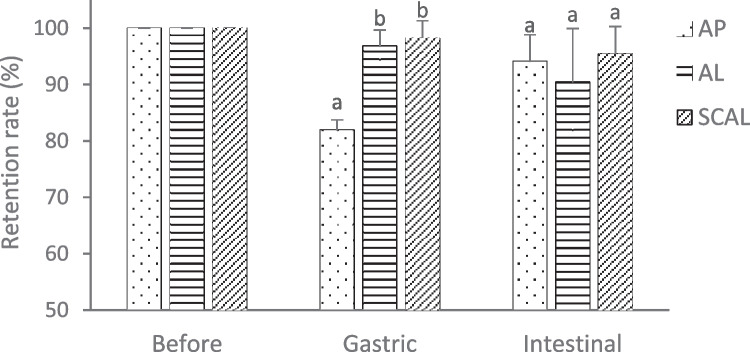
Retention rate of apigenin before and after simulated gastric and intestinal digestion. Different lowercase letters indicate significant difference (p < 0.05) in the same group (n = 3). AP: unencapsulated apigenin; AL: apigenin liposomes; SCAL: spray-dried chitosan-coated apigenin liposomes

The stability of AP against gastrointestinal digestion is vital in ensuring its bioaccessibility, which is the amount of compound released from encapsulating matrix that is made available for intestinal absorption (Angelino et al., 2017). Figure 6 shows the retention rate of AP in the samples after in-vitro simulated gastric and intestinal digestion. After gastric digestion, the retention rate of unencapsulated AP was lower than that of its encapsulated counterparts SCAL and AL (p < 0.05). The results showed that both liposomes and chitosan-coated liposomes reduced the degradation of AP in a highly acidic gastric environment (pH 2). Nevertheless, AP was found to be relatively stable against intestinal digestion (94.08 ± 4.74%) than gastric digestion (81.94 ± 1.80%).

**Fig. 7:**
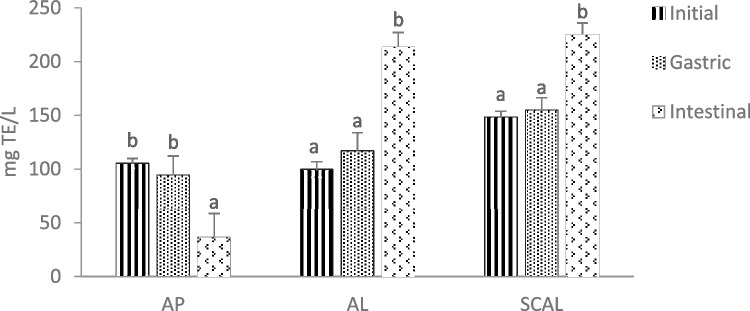
ABTS scavenging activity before and after simulated gastrointestinal digestion. Different lowercase letters indicate significant difference (p < 0.05) in the same group (n = 3). AP: unencapsulated apigenin; AL: apigenin liposomes; SCAL: spray-dried chitosan-coated apigenin liposomes

As shown in Fig. 7, gastric digestion did not significantly affect the antioxidant activity of all samples. However, after intestinal digestion, the ABTS scavenging activity of unencapsulated AP had markedly decreased; in contrast, SCAL and AL had a significant increase in their antioxidant activity (p < 0.05). Previous studies on spirulina hydrolysate liposomes (Mohammadi et al., 2023) and oyster protein hydrolysate liposomes (Sepúlveda et al., 2021) had also reported increased antioxidant activity after intestinal digestion. There are two possible reasons. Firstly, liposomal encapsulation may have preserved the antioxidant activity of AP during the gastrointestinal digestion process. Secondly, as mentioned earlier, phosphatidylcholine of soy lecithin has been reported with antioxidant properties. As pancreatin consists of proteolytic enzymes (trypsin and chymotrypsin), lipase, and amylase (Minekus et al., 2014), the pancreatic lipase may have disrupted the liposomal structure, releasing free phosphatidylcholines that are more readily to scavenge the ABTS radicals, thus leading to the increase of antioxidant activity (Mohammadi et al., 2023).

## Conclusion

In this study, we produced homogenous small unilamellar liposomes encapsulating apigenin at 74.88 ± 5.31% EE from the ethanol injection method without sonication or homogenisation. Chitosan coating at 0.25% w/v could achieve liposomal stability by electrostatic repulsion effect. The results show that the spray drying of chitosan-coated apigenin liposomes is possible at an inlet temperature of 120 ºC and feed rate of 2.5 mL/min to obtain 66.62 ± 3.08% yield. The spray drying process did not affect the concentration of apigenin or the samples’ total phenolic content. SCAL appeared mostly spherical with wrinkles and dents under SEM observation. The aqueous solubility of encapsulated apigenin had significantly improved compared to unencapsulated apigenin, which could facilitate the delivery of apigenin in hydrophilic food matrices. Furthermore, encapsulation of apigenin resulted in higher antioxidant capacity than unencapsulated apigenin due to the synergistic antioxidant potential of soy phosphatidylcholine and chitosan. With chitosan coating, the apigenin liposomes exhibited a prolonged release effect, in which a slower apigenin cumulative release profile was observed compared to the uncoated apigenin liposomes. For in-vitro gastrointestinal stability, SCAL was more stable against highly acidic gastric digestion than the unencapsulated apigenin. The antioxidant capacity of SCAL was not affected after undergoing gastric and intestinal digestion. However, the in-vitro antioxidant capacity of SCAL increased after undergoing gastric and intestinal digestion compared to the unencapsulated apigenin. In conclusion, SCAL could be a promising delivery system for apigenin to be applied in functional foods. In further studies, the stability of SCAL against environmental factors (temperature, pH, oxygen, light) and the application of SCAL in food systems will be examined.

## Data Availability

The data obtained for the publication of this article are available upon reasonable request to the corresponding author.
